# Coinfection with *Rickettsia helvetica* and Herpes Simplex Virus 2 in a Young Woman with Meningoencephalitis

**DOI:** 10.1155/2011/469194

**Published:** 2011-10-19

**Authors:** Kenneth Nilsson, Katarina Wallménius, Carl Påhlson

**Affiliations:** ^1^Section of Clinical Microbiology, Department of Medical Sciences, Uppsala University, 751 85 Uppsala, Sweden; ^2^Section of Infectious Diseases, Department of Medical Sciences, Uppsala University, 751 85 Uppsala, Sweden; ^3^Centre for Clinical Research, Falu Hospital, 791 82 Falun, Sweden

## Abstract

Herpes virus type 2 DNA was detected by PCR in the cerebrospinal fluid in a young woman presenting with headache, stiff neck and pleocytosis, and serological findings consistent with reactivation. Since she was exposed to ticks, Lyme disease and tick-borne encephalitis were excluded. Further investigation in an ongoing project, using PCR and sequencing of the amplified products, showed the presence of *Rickettsia helvetica* in the cerebrospinal fluid. The bacteria were also isolated in Vero cell culture, and microimmunofluorescence confirmed the development of antibodies against *Rickettsia* spp. with predominance of IgM reactivity consistent with recent infection. She was treated with antibiotics and improved rapidly. The patient could easily have been judged to have isolated herpes meningitis. Because Sweden and other European countries are endemic areas for rickettsioses, the paper reaffirms the importance of investigating for the presence of rickettsial infections in endemic areas in cases of meningitis of uncertain aetiology.

## 1. Introduction

Spotted fever rickettsiae (SFR) are small, aerobic, obligate intracellular gram-negative organisms that primarily depend on hematophagous arthropods as vectors and that have a varying ability to cause disease in humans [1, 2]. Nine species of SFR have been described as emerging pathogens in Europe, and in Sweden, both *Rickettsia helvetica *and *Rickettsia felis *have been reported [3–5]. *Ixodes ricinus *ticks are the main vector and reservoir for *R. helvetica*, which has an estimated prevalence in the tick of between 1.7 and 20% in European countries and is to date the only tick-transmitted SFR reported from Sweden [1, 5–7]. Ticks from migratory birds have recently been shown to carry the SFR agents, which probably contributes to the wide geographic distribution of SFR and frequent reports of species found in new locations [8].

Serosurveys of the population in endemic areas in Europe and Asia have identified significant titres of antibodies in a range between 2.6 and 12.8% [9–13]. Infection caused by *R. helvetica *is a recently recognized spotted fever group rickettsial disease. Most patients are diagnosed with a mild febrile illness, sometimes with a rash. However, a more severe presentation as perimyocarditis has been reported, and in one retrospective case, *R. helvetica *has been associated with subacute meningitis [3, 9, 14]. Of the rickettsial diseases, typhus, spotted fever, and Q-fever all may cause central nervous system (CNS) infection, and of the SFR, *R. rickettsii*, *R. conorii*, and *R. japonica *have a well-documented association with meningoencephalitis [15–17]. Also, *R. felis*, whose main reservoir and vector is cat fleas (*Ctenocephalides felis*), has been shown to cause meningitis in two cases, but has so far not been reported in any vector in Sweden [4, 17]. 

Herpes simplex virus 2 (HSV-2) is closely related to HSV-1 and usually causes genital infections and persist latent infection in neurons in the lumbosacral ganglia. One in four adults is infected in Sweden. The virus is spread sexually, but most of the infections are asymptomatic. In rare cases, however, the course of the infection is more complicated and presents with meningitis and headache, sometimes with recurrent episodes (Mollaret's meningitis) [18].

To raise awareness among clinicians of these infections, their similarities, and the possibility of coinfection, we present the first documented case of acute meningoencephalitis, due to *R. helvetica*. 

## 2. Case Presentation

A previously healthy 23-year-old woman, presenting with headache (4-day duration), stiff neck, and photophobia (1-day duration), was hospitalized in July 2010 at the Department of Infectious Diseases, Uppsala University Hospital, Uppsala, Sweden. She had been living in the eastern archipelago during the previous week. There were no proven tick bites, but a small red papule was noted on her left thigh. 

*Clinical examination* revealed no signs of lymphadenopathy or rash, and there were no pulmonary, genital, or abdominal symptoms. Body temperature was 38.5°C at admission but had previously been normal, probably because the patient had used antipyretic tablets. 

*Laboratory tests* showed C-reactive protein at the upper normal limit (11 mg/L), normal peripheral leukocyte cell count (7.2 × 10^9^/L), and normal values for haemoglobin, sodium, and potassium. The cerebrospinal fluid (CSF) revealed pleocytosis (868 × 10^6^ cells/L, of which 820 × 10^6^/L were mononuclear cells), elevated albumin (2209 mg/L), and a slightly lowered blood glucose ratio (<0.5). She was treated for meningitis of unknown aetiology and started on cefotaxime, ampicillin, valacyclovir, and betamethasone intravenously. She improved rapidly with treatment. Blood and CSF bacterial cultures, as well as tests for *Borrelia* and enteroviruses, were all negative. Tick-borne encephalitis serology was consistent with previous vaccination. PCR for HSV-2 in the CSF was positive. HSV-2 IgM in the serum was weakly positive, and HSV-2 IgG was positive in both serum and CSF with a ratio around 20. This was interpreted as evidence of reactivation or an early phase of infection. By coincidence, as a result of an ongoing project, the possibility of rickettsial infection was considered and the investigation revealed a positive direct immunofluorescence test of the CSF which was confirmed by culture and qPCR of the CSF for SFR. The antibiotic treatment was then changed to tablet doxycycline (100 mg pO b.i.d.) and continued for 2 weeks.

The characteristics and laboratory results are summarized in Table 1.

**Figure 1:**
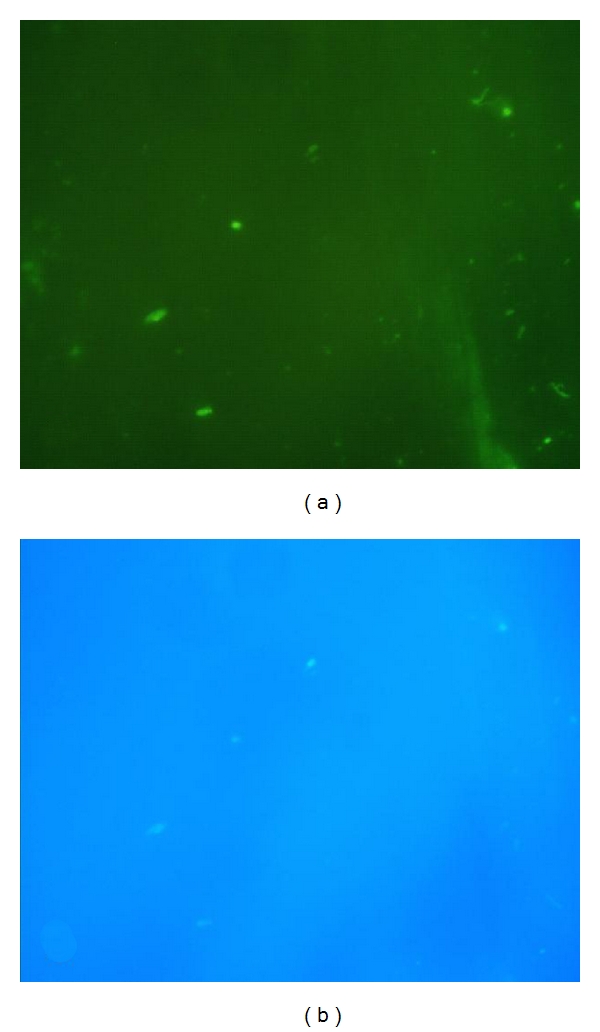
Riskettsial organisms in recently drained CSF; (a) stained with anti-rickettsial antiserum and Alexa Fluor 488 goat anti-rabbit immunoglobulin; (b) the same field of view shows the organisms DNA stained with DAPI in the same location.

**Direct Immunofluorescence on CSF**

after centrifugation, a drop of cerebrospinal fluid, fixed on a microscopic slide and stained with rabbit anti-rickettsial hyper-immunoserum and goat anti-rabbit immunoglobulin, was examined and showed green fluorescent microorganisms of a size and shape corresponding to the *Rickettsia*. As mounting medium VECTASHIELD with DAPI (4′, 6-diamidino-2-phenylindole) (Vector Laboratories, Burlingame, USA) was used according to the manufacturers instruction, showing the same fluorescent organisms positive for bacterial DNA (Figures 1(a) and 1(b)).

**Extraction and PCR**

Bacterial and viral DNA was extracted from a sample of CSF and later from the isolate obtained from Vero cell culture, using the NucliSENS easyMAG automated extraction platform (bioMérieux, Durham, NC, USA) according to the manufacturer's instructions. The spotted fever group of *Rickettsia* was assayed using a genus-specific real-time PCR with probe and primers targeting the *glt*A gene, as previously described [19]. The real-time PCR assays for SFR and HSV were performed in a Rotor-Gene 3000 (Corbett Research, Sydney, Australia) using LC Taqman Master kit (Roche, France) and were positive for both HSV-2 and SFR. Reagent controls, containing no DNA, showed no amplification. The Ct values for SFR were between 34.86 and 37.79, indicating less than ten copies per reaction. This corresponds with the detection limit by direct microscopy where it is possible to detect 10^4^ bacteria/mL.

The SFR positive samples were further analysed using nested PCR assays that amplify the 17-kDa, *omp*B and *glt*A genes, as previously described [20–22]. Conventional and nested PCR were performed in a DNA thermal cycler (Gene Amp PCR System 9700, PE Applied BioSystems, USA), and expected fragment sizes were confirmed using gel electrophoresis (1% agarose, 0.01% ethidium bromide). Confirmation of fragment size was based on a standard DNA molecular weight marker (Fermentas, Helsingborg, Sweden). As a negative control, sterile water was included in each amplification trial. Purified DNA of *R. helvetica* originally isolated from an *I. ricinus *tick was used in these assays as the positive control [7]. Direct cycle sequencing analysis of both strands of the amplicons was performed using an automatic ABI 3130 Genetic Analyzer (Applied Biosystems, Tokyo, Japan). For species identification, pair-wise similarities to and differences from other rickettsiae in the spotted fever group were examined using Blast analysis. Multiple sequence alignments were conducted using BioEdit version 7.0.9 and ClustalW. The obtained sequences in nested and conventional PCR of partial regions of the 17 kDa, *omp*B, and *glt*A genes were 169 bp (17 kDa), 222 bp (*omp*B), and 716 bp (*glt*A), respectively. Analyses of these amplicons shared 99-100% similarity with the corresponding gene sequences of *R. helvetica* (GenBank accession numbers GU827073 (17 kDa), AF123725 (*omp*B), and U59723(*glt*A)), with significant nucleotide differences from the other rickettsiae of the spotted fever group.

**Rickettsial Culture**

100 *μ*L of CSF was cultured in each of two 25 cm^2^ flasks onto confluent monolayers of Vero cells. After inoculation, the cell culture was incubated in Eagle's minimal essential medium, containing 10% fetal calf serum and 1% L-glutamine in a humid chamber in 5% CO_2_, at 32°C. All reagents and cell lines were checked weekly for rickettsial growth and to exclude other bacterial contamination. Detection of rickettsial growth in the cell culture was monitored using a direct immunofluorescence assay of cells collected after centrifuging the medium and attached to a microscope slide. The preparation was stained with rabbit anti-rickettsial hyperimmune serum and goat anti-rabbit IgG (H+L-chain) immunoglobulin (Alexa Fluor 488) (H+L-chain) conjugate (Invitrogen, Carlsbad, CA, USA) as secondary antibody. As mounting medium VECTASHIELD with DAPI (Vector Laboratories, Burlingame, USA) was used. After 3 weeks, intracellular bacteria were observed in the cells and rickettsial DNA was verified by real-time PCR [19, 23].

**Serology**

*HSV serology* was performed in a Behring ELISA processor III and 2000, respectively, according to the manufacturer's instructions (Siemens, Diagnostic Products, Marburg, Germany). HSV IgG (AE/mL) was 67 (positive) in CSF and 1900 (positive) in serum. HSV IgM (OD) was not detected in CSF and was 0.37 (weakly positive) in serum.

*Immunoflourescent detection (IFA)* of antibodies to *R. helvetica* in inoculated Vero cells. An aliquot of infected Vero cell medium was supplemented with 10% yolk sac solution, applied to microscope slide wells, dried, fixed in acetone, and incubated with serial dilutions of serum as previously described [13]. As positive controls, a serum sample from a patient with proven infection with *Rickettsia conorii *with end-point IgG titres of 1 : 160, provided by the Swedish Institute for Infectious Disease Control, and serum from another patient with proven end-point IgG titres of 1 : 80 to *R. helvetica *were used. IgG and IgM antibodies were detected using fluorescein isothiocyanate-conjugated (FITC) *γ*- and Mu-chain, respectively, specific polyclonal rabbit anti-human IgG and IgM (ref.: F0202 and F0203; Dako, Denmark). The IgM antibodies were examined after a pretreatment procedure with rheumatoid factor adsorbent (Immunkemi, Stockholm, Sweden) to remove complex bound IgG antibodies. The IFA results are summarized in Table 1. Predominant IgM reactivity to *R. helvetica* antigen and the spotted fever group antigen was demonstrated in the acute and convalescent phase sera. All negative controls were negative.

## 3. Discussion

The patient in this paper presented with symptoms of increasing headache, neck stiffness, and fever after travelling to the eastern archipelago of Sweden in the previous week. Laboratory investigations showed a CSF pleocytosis, with mononuclear predominance, a positive PCR test for HSV-2, and HSV-2 serology indicating reactivation or early infection, resulting in a diagnosis of HSV-2 meningitis and appropriate treatment. There were no classic tick bite marks or a rash suggestive of rickettsial infection. Due to possible tick exposure, and as a result of an ongoing *Rickettsia* project, this patient was also included for investigation, which verified the presence of *R. helvetica *in the CSF.

All the available diagnostic methods confirmed a rickettsial infection. Direct immunofluorescence was a useful tool in this case, due to the white blood cell elevation in the CSF. As with other similar assays, if the bacterial load is low in the CSF, the result may be negative (Figures 1(a) and 1(b)). Furthermore, it requires technical expertise for reliable interpretation.

Real-time PCR analysis of CSF provides the ability to detect and diagnose early stages of rickettsial infections in CNS but a conventional PCR is helpful to further amplify and sequence the product for species identification. In Sweden, another spotted fever *Rickettsia*, *R. felis*, has also been shown to cause meningitis recently. Distinguishing between these two rickettsiae requires primer design encompassing a sequence sufficient for species differentiation [4].

Serological testing confirmed the presence of significant titres of anti-rickettsial antibodies, predominantly IgM with a higher titre in the acute-phase serum and decreasing during convalescence. The development of an IgG titre was more moderate, but it is reported that, in primary infections, individuals who have received antibiotic therapy initiated during the first week after disease onset showed strong IgM responses without pronounced increases in IgG [24]. The present findings demonstrate the importance of including an IgM assay to obtain a reliable serological assessment and better chance of early diagnosis.

We also isolated the rickettsial organism. In this case, the conditions were optimal, because the delay between the time of sample collection and inoculation onto shell vials was only a matter of hours. Previous studies have shown that all cultures of samples inoculated later than the day of sampling and held at 4°C or room temperature were negative [25]. Isolation therefore requires access to specialized laboratory facilities within a reasonable distance.

For patients without rash or eschar, rickettsial diagnosis is not easy and cannot be definitively established on epidemiological, clinical, and standard laboratory criteria. A diagnostic score to help the physician assess the diagnosis of SFR has been previously described for Mediterranean spotted fever, African tick bite fever, and lymphangitis-associated rickettsiosis, but not for other less established rickettsial diseases [26]. The diagnosis is established when laboratory findings and epidemiological, clinical, bacteriological, and serological criteria give a score equal to 25 or higher. In this case, using the same criteria, isolation of the bacterium and the serological findings provided a score well above 25. The utility of the proposed criteria should be studied further for other species of rickettsial diseases. 

The standard treatment regimen for SFR consists of 200 mg doxycycline (orally or intravenously), daily for 7–14 days depending on the clinical course [27]. Most patients will improve within the first 24 h after treatment begins, which has led to the proposal of shorter regimens, but treatment should at least be continued until the patient is afebrile for 24 h. Obviously the patient was also infected with HSV-2. She had no previous history of recurrent episodes of severe headache, and the serological findings were suggestive of reactivated HSV-2 infection, which was probably asymptomatic in her case. It is possible that the rickettsial infection reactivated her HSV-2 infection but the opposite is conceivable as well as simultaneous infection by both agents at the same time. Similar co-infection with *R. japonica *and EBV, another latent virus, has been reported in a case with hepatic failure [28]. There are similarities between the classic symptoms of Mollaret's meningitis and a rickettsial infection, suggesting that as long as the cause of Mollaret's meningitis is unknown, it might be of interest to investigate for SFR in cases who may have been exposed to *Rickettsia*.

Early diagnosis of rickettsial infection is important, and though Sweden, like other European countries, is an endemic area for *R. helvetica*, it is of importance that clinicians routinely consider rickettsial infections, besides other agents, in patients with fever or meningitis residing in or travelling to endemic areas. Since rickettsial infections are eminently treatable, the availability of appropriate and timely diagnostics is essential to optimize the clinical outcome of those affected. 

## Figures and Tables

**Table 1:** Summary of the disease history and laboratory findings. R.h.: *R. helvetica*; doxy: doxycycline.

*Characteristics*	
Sex, age in yrs	F, 23
Month	July
Fever, temp °C	38.5
Headache	Yes
Stiff neck	Yes
Photophobia	Yes
Treatment	doxy
Outcome	Cured

*Lab results *	
C-reactive prot, mg/L	11
WBC count, g/L	7.2

*CSF results *	
CSF cells (×10^6^/L) total	868
Mono	820
Proteins (mg/L)	2209
CSF/blood glucose ratio	0.46

*Serology *	
Acut phase/s	
Ric-MIF IgG	<32
Ric-MIF IgM	2048
CSF Ric-MIF IgG	<32
Ric-MIF IgM	<1/16
Convalescent phase/s	
Ric-MIF IgG	64
Ric-MIF IgM	1024
Borrelia (IU/mL) serum	Neg
(Index) CSF	Neg

*Isolation *	R.h
